# Hemozoin‐induced activation of human monocytes toward M2‐like phenotype is partially reversed by antimalarial drugs—chloroquine and artemisinin

**DOI:** 10.1002/mbo3.651

**Published:** 2018-06-07

**Authors:** Deepali Bobade, Ashwin V. Khandare, Mangesh Deval, Padma Shastry, Prakash Deshpande

**Affiliations:** ^1^ National Centre for Cell Science (NCCS) Pune India

**Keywords:** artemisinin, chloroquine, hemozoin, M2 monocytes, malaria

## Abstract

*Plasmodium falciparum* malaria is the most severe form of malaria with several complications. The malaria pigment‐hemozoin (Hz) is associated with severe anemia, cytokine dysfunction, and immunosuppression, thus making it an interesting target for developing new strategies for antimalarial therapy. Monocytes (MO) in circulation actively ingest Hz released by *Plasmodium* parasites and secrete pro‐ and anti‐inflammatory cytokines. M1 and M2 types represent the two major forms of MO/macrophages (MQ) with distinct phenotypes and opposing functions. Imbalance in the polarization of these types is reported in many infectious diseases. Though the association of Hz with immunosuppression is well documented, its role in activation of MO in context of M1/M2 phenotypes remains to be addressed. We report here that natural Hz drives human MO toward M2‐like phenotype as evidenced by the expression of M2 signature markers. Hz‐fed MO showed elevated transcript and secreted level of IL‐10, CCL17, CCL1, expression of mannose‐binding lectin receptor (CD206), and arginase activity. Hz attenuated HLA‐DR expression, nitric oxide, and reactive oxygen species production, which are the features of M1 phenotype. Our data also implicate the involvement of p38 MAPK, PI3K/AKT, and NF‐κB signaling pathways in skewing of Hz‐fed MO toward M2‐like type and suppression of mitogen‐stimulated lymphocyte proliferation. Importantly, antimalarial drugs—chloroquine and artemisinin—partially reversed activation of Hz‐induced MO toward M2‐like phenotype. Considering the limitations in the current therapeutic options for malaria, we propose that these drugs may be re‐examined for their potential as immunomodulators and candidates for adjunctive treatment in malaria.

## 1 INTRODUCTION

Malaria is one of the leading causes of morbidity affecting around 212 million individuals and 4, 29,000 deaths in endemic regions according to WHO 2015. *Plasmodium falciparum* causes the most severe form of malaria with complications that include cerebral malaria, pulmonary edema, acute renal failure, or severe anemia (Buffet et al., 2011; Das, 2008; Medana & Turner, 2006; Mohan, Sharma, & Bollineni, 2008)*. P. falciparum* malaria is frequently associated with immunosuppression and increased secondary infections (Hu, 2013; Ihekwereme, Esimone, & Nwanegbo, 2014; Orf & Cunnington, 2015; Troye‐Blomberg & Perlmann, 1988). Immunosuppression enhances parasitemia and reduces immune responses to not only malaria parasite proteins but other pathogens as well. Accumulating evidence suggests that a combination of parasite and host factors is involved in the pathogenesis of severe malaria.

During the intra‐erythrocytic stages, *P. falciparum* parasites digest hemoglobin in the food vacuole, resulting in the production of potentially toxic heme metabolites (Francis, Sullivan, & Goldberg, 1997), which the parasite detoxifies by converting it to an insoluble crystal called malarial pigment or hemozoin (Hz) (Arese & Schwarzer, 1997). During schizogeny, Hz is released into blood and avidly phagocytosed by human phagocytic cells including monocytes (MO) (Shio, Kassa, Bellemare, & Olivier, 2010). As a result of Hz uptake, several functions of MO such as repeated phagocytosis (Schwarzer et al., 1992), bactericidal abilities (Fiori et al., 1993), oxidative burst, MHC Class II expression, antigen presentation (Scorza, Magez, Brys, & De Baetselier, 1999), and maturation to dendritic cells (Urban & Todryk, 2006) are severely affected. MO actively respond to ingested natural Hz by secretion of pro‐ and anti‐inflammatory cytokines, TNF‐α, IL‐1β, IL‐1RA, IL‐10, IL‐8, and chemokines, CCL2, CCL3 CCL4, CXCL1, CXCL2, CXCL3 and CXCL5 (Deshpande & Shastry, 2004; Giribaldi et al., 2010). Hz stimulates IL‐1β, TNF‐α, and CCL3 production via NF‐κB and p38 MAPK kinase in human MO (Polimeni et al., 2012). Purified Hz isolated from parasitized RBCs stimulates TLR9 signaling and induces production of cytokines—TNF‐α, IL‐6, IL‐12p40, CCL2 in MYD88‐dependent manner in murine spleen and dendritic cells (Coban et al., 2005). In murine MQ cell line B10R, NF‐κB has been shown to be involved in chemokine production of CCL2, CCL3, CCL4, and CXCL2 induced by Hz (Jaramillo, Godbout, & Olivier, 2005). Hz‐induced activation of NOD‐like receptor containing pyrin domain 3 inflammasome and IL‐1β production is mediated through Src kinase Lyn and the tyrosine kinase Syk pathways in mice (Olivier, Van Den Ham, Shio, Kassa, & Fougeray, 2014; Shio et al., 2009).

MO are heterogeneous innate immune cells that play a pivotal role in primary response to pathogens, tissue homeostasis, inflammation, resolution, and repair (Shi & Pamer, 2011). MO are highly plastic and polarize toward M1 phenotype in the presence of inflammatory environment such as LPS and IFNγ, while the anti‐inflammatory M2 phenotype is driven by TH2 cytokines IL‐4, IL‐13, IL‐10, and TGF‐β. IL‐12‐producing M1 phenotype is associated with antiparasitic and tumor‐resistance capabilities, and IL‐10‐producing M2 phenotype is related to wound healing, immune regulation, and resolution of inflammation (Mantovani et al., 2004; Martinez & Gordon, 2014; Sica & Mantovani, 2012). M1 and M2 MO have an indirect role in the activation of Th1 and Th2 responses, respectively (Mantovani et al., 2004). A study by Murray et al. (2014) described different types of MQ phenotypes generated by different activating agents and referred to them as M(IL‐4), M(Ig), M(IL‐10), M(GC), M(IFNγ), and M(LPS); however, a phenotype may not fall into specific categories but may show a spectra related to particular phenotype. The M2 phenotype displays high expression of mannose‐binding receptor CD206, high arginase activity, and low nitric oxide (NO) production (Gordon, 2003; Kobayashi et al., 2010; Mosser, 2003; Tsuchimoto et al., 2015). The M2 type MO are divided into M2a, M2b, and M2c subtypes that produce specific chemokines CCL17, CCL1, and CXCL13, respectively (Edwards, Zhang, Frauwirth, & Mosser, 2006; Mantovani et al., 2004; Sironi et al., 2006).

Multiple signaling pathways including JAK/STAT, PKC/ERK, and PI3K/AKT/mTOR are involved in the maintenance of M2 phenotype. The pathways may act individually or in combination to drive MO toward M2 polarization (Gordon & Martinez, 2010; Martinez & Gordon, 2014).The p38 MAPK and PI3K‐AKT pathway mediated IL‐4‐induced M2a polarization in mice model (Jimenez‐Garcia, Herranz, Luque, & Hortelano, 2015). Notch1 signaling via NF‐κB, p38 MAPK, and AKT pathways has been demonstrated in M2b polarization of murine lupus mouse model (Zhang, Xu, & Xiong, 2010). Recent studies have enhanced our understanding and importance of phenotypic changes in MO by modulating immune responses in several infectious diseases (Buchacher, Ohradanova‐Repic, Stockinger, Fischer, & Weber, 2015; Kobayashi et al., 2010; Nakamura, Ito, Kobayashi, Herndon, & Suzuki, 2015; Tsuchimoto et al., 2015) and highlighted their potential for development of new therapeutics. Phenotypic changes in MO have been reported in parasitic diseases such as leishmaniasis, filariasis, and trypanosomiasis (Babu et al., 2009; Cabalén et al., 2016).

While accumulating experimental and clinical data support the significant contribution of Hz in immunosuppression, its role in driving the MO toward specific MO phenotypes and the implications therein remains to be unraveled. In the present study, we aimed to (a) investigate the effect of Hz in the activation of human MO toward M2 phenotype, (b) examine the signaling pathways involved in the process, and (c) explore the potential of antimalarial drugs—chloroquine (CHQ) and artemisinin (ART)—in reversion of Hz‐driven activation of MO.

## 2 MATERIALS AND METHODS

### 2.1 Chemicals and reagents

Sterile tissue culture plastics were purchased from corning. RPMI 1640 media was purchased from Sigma. The parasite and MO cultures were ensured to be mycoplasma free and endotoxin low (<0.125). Inhibitors SB203580 (SB), parthenolide (PAR), LY294002 (LY), and drugs CHQ diphosphate and ART were purchased from Sigma.

### 2.2 Parasite culture and natural Hz isolation

Natural Hz from *P. falciparum* was isolated as described previously with slight modifications (Barrera et al., 2011; Bujila et al., 2016; Schwarzer et al., 1992; Skorokhod, Alessio, Mordmuller, Arese, & Schwarzer, 2004). Briefly, *P. falciparum* (3D7 strain; MR4/ATCC, mycoplasma free) culture was maintained in candle jars at 37°C (Trager & Jensen, 1976). Parasites were cultured in O‐positive RBCs at 5% hematocrit in RPMI 1640 medium (Sigma) supplemented with 25 mM HEPES, 4.5 g/L glucose and 2.1 g/L NaHCO_3_, 0.5% AlbuMAX II (Thermo Fisher Scientific), and gentamicin (50 mg/ml). The medium was replenished every 24 h. Parasite cultures with >25% rings were synchronized by 5% aqueous sorbitol and cultured for 50 h until the rupture of mature schizonts and release of Hz. Culture supernatants containing Hz were carefully aspirated and collected and centrifuged, and the blackish‐brown Hz‐containing pellet was washed three times with sterile ice cold PBS (pH 7.2). Pigment isolated from different batches of parasite cultures was pooled, aliquoted, and stored in PBS at −20°C. The total heme content in isolated Hz was determined as previously described (Polimeni et al., 2012). Hz isolated from the supernatants of synchronized parasite cultures after schizont rupture is mostly devoid of parasite material. *P. falciparum* DNA was not detected in natural Hz (at concentration used in our experiments) on agarose gel (Supporting Information Figure S1).

### 2.3 Isolation of MO (CD14^+^)

The study for use of human blood MO was approved by the ethics committee of National Centre for Cell Science, Pune, India. Buffy coats were obtained from healthy donors recruited for blood collection at government‐recognized blood bank, Pune. Informed consent from donors was not essential as discarded buffy coats obtained after plasmapheresis were used. Peripheral blood mononuclear cells (PBMCs) were isolated from buffy coats by Histopaque 1077. CD14^+^ MO were enriched from PBMCs by positive selection using magnetic beads (MACS) (Miltenyi Biotec, Germany), according to the manufacturer's instructions. The purity of MO obtained was 85%–90% CD14^+^ and viability >95% (Supporting Information Figure S2). Adherent MO for phagocytosis was obtained as previously described with some modifications (Polimeni et al., 2012). Briefly, 2 × 10^6^ MO/2 ml was seeded per well in six‐well tissue culture plates and incubated for 1 h at 5% CO_2_ in RPMI 1640 medium supplemented with 25 mM HEPES, 2 mM glutamine, 100 mg/ml streptomycin, 100 units/ml Penicillin G, and 10% fetal bovine serum (Sigma St Louis, MO). Cells were washed with RPMI 1640 medium, and the adherent MO obtained was incubated at 37°C overnight. The adherence step applied after MACS and before phagocytosis further enriched the MO population to contain >95% MO. Culture medium was replaced with fresh medium before the start of experiments.

### 2.4 Phagocytosis of Hz and *in vitro* cell culture experiments

Phagocytosis of Hz and latex particles was carried out as previously described (Polimeni et al., 2012). Parasite Hz (50 μg/ml) and latex beads (0.114 μm diameter; 10 μl of a 100‐fold dilution of the 2.5% V/V) were exposed to 10^6^ MO. The dose of pigment used was based on Hz amounts detected during *P. falciparum* infection and previous *in vitro* and *in vivo* studies (Deshpande & Shastry, 2004; Jaramillo et al., 2005). The latex beads were used as phagocytic control (Giribaldi et al., 2010). The plates were incubated in 5% CO_2_ incubator at 37°C for 2 h to allow phagocytosis. Un‐phagocytized Hz and beads were removed by media washes, and the cells were incubated for indicated time intervals. In cell signaling experiments, the MO were pretreated with different pharmacological pathway inhibitors, SB203580 (10 μM), parthenolide (10 μM), and LY294002 (10 μM) 1 h prior to phagocytosis. Antimalarial drugs—ART (20 μM) and CHQ (20 μM)—were added 2 h after exposing the MO to Hz pigment.

### 2.5 Flow cytometry

The MO fed with Hz and latex beads were collected after 24 h by gentle scraping in 5 mM EDTA, washed with 1× PBS (without Ca^−^/Mg^−^), supplemented with 2% FBS on ice. The cells were passed through cell strainer (40 μM) and incubated with human TruStain FcX blocking reagent (Biolegend) to inhibit nonspecific binding of antibodies. The cells were stained with PE anti‐human CD14 (M5E2) and FITC anti‐human HLA‐DR (Clone G46‐6), APC anti‐human CD206 (19.2) (Biolegend), and respective isotypes (Biolegend) for 30 min at 4°C. Cells were washed with 1× PBS (without Ca^−^/Mg^−^) supplemented with 2% FBS, and data were acquired on BD FACS Canto II (BD Biosciences) and analyzed using FACS DIVA software and FlowJo software (BD Biosciences). All parameters measured were based on SSC versus MO‐specific CD14^+^ gated cells.

### 2.6 RNA isolation and cDNA synthesis

The MO fed with Hz/latex were collected after 12 h and stored in Trizol reagent (Life Technologies, Inc., Gaithersburg, MD) at −80°C until RNA isolation. Total RNA was extracted using the Purelink RNA kit (Ambion, USA) following the manufacturer's protocol of Trizol plus Purelink columns. The purified total RNA was resuspended in RNase‐free water and quality was analyzed using Nanodrop (ND‐1000 spectrophotometer). 600 ng of RNA was reverse transcribed with ImProm‐II^™^ Reverse Transcription System (Promega) as per manufacturer's instructions.

### 2.7 Quantitative real‐time polymerase chain reaction (qPCR)

qPCR analysis of mRNA expression of M1 and M2 phenotype‐specific marker genes in Hz and latex fed MO was performed using Power syber green (Thermo Fisher Scientific) and gene‐specific primers. The primers related to M1/M2 phenotype for real‐time PCR analysis were designed using NCBI primer 3 Software and were custom synthesized from Sigma and IDT technologies. Oligo sequences are listed in Supporting Information Table S1. The cycling parameters were as follows: Initial denaturation at 95°C for 10 min to activate the AmpliTaq Gold DNA polymerase followed by 95°C for 15 s, 60°C for 60 s, and then 72°C for 30 s for a total of 40 cycles. The comparative threshold cycle (*C*_t_) value for GAPDH was used to normalize loading variations in the real‐time PCR. The extent of mRNA expression for a given condition was represented by the relative value to the lower DDCT between those estimated for the different conditions: the control DDCT value was fixed at 1.0. The specificity of PCR was confirmed by melting curve analysis. Real‐time PCR was done on thermocycler Applied Biosystems 7300 (Applied Biosystems).

### 2.8 ELISA

Cytokines TNF‐α, IL‐1β, IL‐6, IL‐10, and IL‐12p70 and chemokines, CCL1, CCL17, and CXCL13 levels were quantified in the cell free‐culture supernatants using ELISA kits according to manufacturer's instructions. Human ELISA kits for CCL1 and CXCL13 were purchased from R&D Biosystems; CCL17, IL‐12, TNF‐α, and IL‐6 were purchased from Biolegend; and IL‐10 and IL‐1β were purchased from BD Biosciences.

### 2.9 Western blot analysis

The MO fed with Hz were washed with PBS and lysed with equal volumes of RIPA buffer containing 1× protease inhibitor cocktail on ice and then centrifuged for 10 min at 16,000 × g at 4°C. Lysates with equal amounts of protein were separated by 10% SDS‐PAGE and then transferred onto PVDF membrane (Millipore, Bedford, MA, USA), blocked with 5% Milk in TBS containing 0.1% Tween 20, and incubated overnight at 4°C with primary antibodies. Phospho‐AKT, phospho‐P38, phospho‐p65 (NF‐κB), total p65 (NF‐κB) were purchased from Abcam; total P38 from Santa Cruz Biotechnology; AKT from Cell signaling technology, IκBα from Santa Cruz Biotechnology, and GAPDH from Sigma. The membranes were exposed to HRP‐labeled anti‐rabbit IgG secondary antibodies (1:5,000) (BioRad) for 1 h at RT and detected by enhanced chemiluminescence detection system and visualized using GE image quant 4000 chemiluminescence system (GE Healthcare Lifesciences).

### 2.10 Lymphocyte proliferation assay

Adherent MO were prepared and exposed to Hz and latex as described above, and autologous lymphocytes previously collected were added in the ratio of 1:4–1:8. The cells were incubated for 72 h in a total volume of 200 μl of complete media (RPMI + 10%FCS) with 5 μg/ml of PHA‐L (Sigma) mitogen. Cells were pulsed with 1 μCi/well [methyl‐^3^H] Thymidine (Amersham) for 18 h before harvesting. The CPM counts were recorded in liquid beta‐scintillation counter (Packard). (^3^H) Thymidine incorporation was measured as CPM and compared with untreated cells.

### 2.11 Arginase activity assay

Arginase activity was determined by colorimetric assay (Sigma) according to the manufacturer's instructions. Briefly, MO fed with Hz and latex were lysed with 10 mM Tris–HCl, pH 7.4 containing 1 μM pepstatin A, 1 μM leupeptin, and 0.4% (w/v) Triton X‐100. The samples were centrifuged at 13,000 × g for 10 min to remove insoluble material. The samples were incubated with substrate buffer containing arginine buffer and manganese (Mn) solution for reaction to occur for 2 h at 37°C along with appropriate sample blank and urea standard in separate wells. The reaction was terminated by adding urea reagent and incubation for 1 h. The absorbance was recorded at 430 nm and arginase activity was calculated as units/L.

### 2.12 NO estimation

The NO production in the culture supernatants of Hz and latex fed MO was measured as stable end product of NO, that is, nitrite using the Griess reagent. Briefly, 100 μl of cell culture supernatant was added to 96‐well plate in triplicates, followed by 50 μl sulphanilamide and 50 μl *N*‐1‐napthylethylenediamine dihydrochloride. The plate was incubated in dark for 15 min at 37°C. Absorbance at 540 nm was measured by microplate reader, and nitrite concentration was estimated using a sodium nitrite standard curve. RPMI1640 media without phenol red dye was used for this assay.

### 2.13 ROS detection assay

Reactive oxygen species (ROS; including superoxide, hydrogen peroxide, and other reactive oxygen intermediates [ROI]) was estimated by H_2_O_2_‐sensitive probe H_2_DCFDA (Thermofisher scientific) by flow cytometry. Briefly, Hz and latex fed MO were incubated with H_2_DCFDA (10 μg/mL) and PE‐anti‐human CD14 at RT for 20 min in the dark. PE‐labeled anti‐human CD14 was used to gate MO. Fluorescent DCF in the MO emitting green fluorescence and proportional to intracellular ROS was acquired on BD FACS Canto II, and data were analyzed using FACS DIVA software.

### 2.14 Statistical analysis

All values (expressed as *M* ± SEM) were obtained from three or more independent experiments. Comparison between groups was done with the one‐way ANOVA (Bonferroni correction). Statistical comparisons were made as indicated, significance levels: *p < 0.05, **p < 0.01, ***p < 0.001. GraphPad Prism6 software was used for statistical analysis.

## 3 RESULTS

### 3.1 Hz induces M2‐like phenotype in peripheral blood‐derived‐human MO

**Figure 1:**
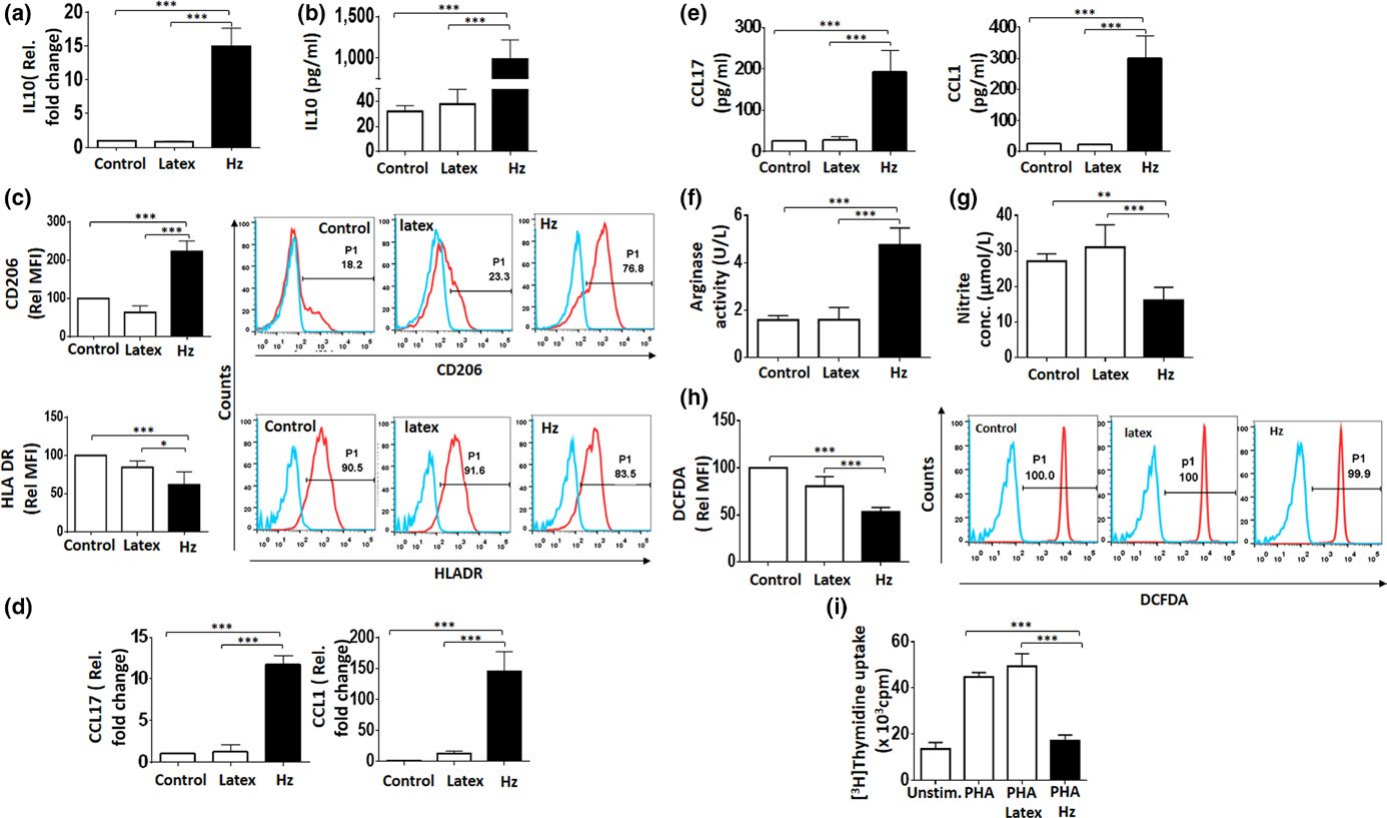
Hemozoin (Hz) induces M2 (M2a and M2b) like phenotype in peripheral blood‐derived human monocytes (MO). (a) Hz‐fed MO were assessed for expression of IL‐10 (M2) transcript (12 h) after exposure by qPCR. GAPDH was used as an endogenous control. (b) Levels of the secreted IL‐10 cytokine in culture supernatant (24 h) post Hz phagocytosis by ELISA. (c) Relative mean fluorescent intensity (MFI) of CD206 (M2) and HLA‐DR (M1) after Hz exposure (24 h) as determined by flow cytometry. Representative overlays depicting treated cells with percentage positive MO. (d) Hz‐fed MO (12 h) assessed for phenotypic markers of M2 subtypes; CCL17 (M2a) and CCL1 (M2b) transcripts were detected by qPCR. GAPDH was used as an endogenous control (e) secreted chemokine CCL17 (M2a) and CCL1 (M2b) levels in culture supernatant (24 h) post Hz phagocytosis by ELISA. (f) Arginase (M2) activity in cell lysates from Hz‐fed MO (24 h) as determined by colorimetric assay. (g) Nitric oxide (M1) release in culture supernatants from Hz‐fed MO (24 h) as quantified by Griess assay. (h) Intracellular reactive oxygen species (ROS) (M1) monitored by flow cytometry as a measure of DCF fluorescence post DCF‐DA probe staining. The relative MFI of DCFDA and representative overlays with percentage positive MO for ROS are shown. (i) Suppression of lymphocyte proliferation as assessed by (^3^H) Thymidine incorporation in Hz‐fed MO. Latex beads were used a phagocytosis control. Results are *M* ± SEM from at least three independent donors. Significance levels: *p < 0.05, **p < 0.01, ***p < 0.001 in comparison with the control and latex as determined by one‐way ANOVA (Bonferroni test)

The effect of Hz on activation of MO toward the M2 phenotype was examined in CD14^+^ sorted cells and analyzed for a panel of M1‐ and M2‐associated markers. IL‐10, a well‐characterized anti‐inflammatory cytokine implicated in M2 polarization, was elevated at transcript level following Hz exposure (Figure 1a). Consistent with our previous work (Deshpande & Shastry, 2004), a strong induction of secreted and cellular IL‐10 was observed (Figure 1b and Supporting Information Figure S3a). The transcript and secreted level of IL‐12p70 was assessed in Hz‐fed MO, and treatment with LPS + IFNγ was used as positive control. Interestingly, Hz phagocytosis did not alter the IL‐12p70 level. As expected, the production of IL‐12 was increased in MO exposed to LPS + IFNγ (Supporting Information Figure S3b,c). M1 phenotype is also induced by microbial products through TLR ligands (Murray & Wynn, 2011; Murray et al., 2014; Wang, Liang, & Zen, 2014). Therefore, additional experiments were conducted to examine TLR engagement in Hz‐driven MO activation. MO exposed to natural Hz were analyzed by flow cytometry and western blotting for expression of TLR9. β‐Hematin (sHz) was used as a reference for flow cytometry analysis (Coban et al., 2005). As shown in Supporting Information Figure S4a,b, natural Hz had no effect on TLR9 expression, suggesting that it might not be involved in Hz‐mediated MO activation. In this study, MO fed with Hz displayed strong upregulation of mannose‐binding lectin receptor (CD206), a M2‐associated surface marker with increase in percent positive cells and MFI in comparison with controls (Figure 1c). HLA‐DR, a M1 phenotype‐related marker, was significantly down‐regulated as observed by decrease of MFI in response to Hz exposure (Figure 1c). Recent reports employing differential gene expression and chemokine profiles have ascribed three subtypes in the M2 phenotype. To identify the M2 subtypes generated following Hz phagocytosis, we assessed the expression of subset‐specific chemokines—CCL17 (M2a), CCL1 (M2b), and CXCL13 (M2c). Hz‐fed MO robustly elevated transcript level of CCL17 and CCL1 (Figure 1d). Also, a significant increase in secreted chemokines was observed for CCL17 and CCL1 in supernatants of Hz‐fed MO (Figure 1e). In addition, Hz phagocytosis elevated transcript level and secretion of M2b‐associated cytokines—TNF‐α, IL‐1 β, and IL‐6 (Supporting Information Figure S5a,b). The amount of Hz in the range of 25–75μg/ml significantly elevated the secreted levels of IL‐10, CCL17, CCL1, and surface expression of CD206 in MO (Supporting Information Figure S5c). Interestingly, CXCL13 and CD163 (markers related to M2c phenotype) were unaltered under similar experimental conditions (Supporting Information Figure S6).

MO polarization endows subtypes with specific biochemical and functional properties. M1 MO are characterized by elevated production of NO and ROI that are essential for clearance of pathogens. Conversely, M2 MO lack the ability to generate oxidative intermediates, synthesis of ornithine and polyamines through the arginase pathway. We found that Hz‐fed MO robustly elevated arginase activity (Figure 1f) and significantly reduced the NO (Figure 1g) and ROS levels (Figure 1h) compared with untreated and latex‐ingested MO controls. Furthermore, Hz‐fed MO displayed a significant suppression of PHA‐stimulated lymphocyte proliferation when compared with control and latex‐ingested MO (Figure 1i), indicating an immunosuppressive response. Collectively, these results demonstrate that the malarial pigment–Hz induced M2‐like phenotype and more specifically toward M2a‐ and M2b‐like subtypes in human MO.

### 3.2 Hz facilitates the M2‐like phenotype via p38 MAPK, PI3K‐AKT, and NF‐κB pathways

**Figure 2:**
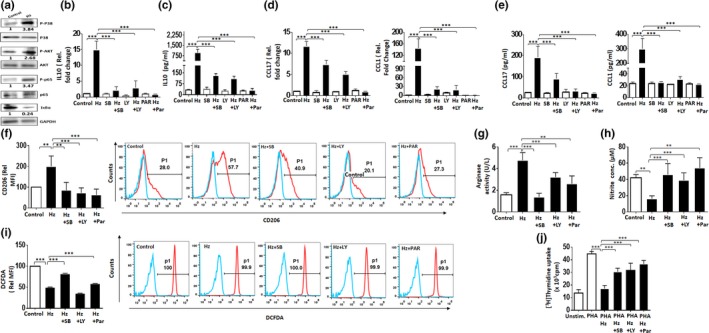
Hemozoin (Hz) facilitates the M2‐like phenotype *via* p38 MAPK, PI3K‐AKT, and NF‐κB pathways. (a) Western blot analysis of whole‐cell lysates from control and Hz‐fed monocytes (MO) (2 h) for total and phosphorylated levels of p38 MAPK, NF‐κB p65, IκBα, and AKT. Fold change obtained from densitometric analysis of phospho‐p65‐NF‐κB, AKT, IκBα, and p38, normalized with GAPDH are indicated above blots. (b) The effect of p38‐MAPK pathway inhibitor SB203580 (SB), NF‐κB pathway inhibitor parthenolide (PAR), and PI3k‐AKT pathway inhibitor LY294002 (LY) on Hz‐induced IL‐10 transcript (12 h) in Hz‐fed MO. GAPDH was used as an endogenous control. (c) Secreted IL‐10 post Hz exposure (24 h) from culture supernatants (24 h) in the presence of SB, PAR, and LY as estimated by ELISA. (d) Expression of Hz induced CCL17 (M2a) and CCL1 (M2b) in the presence of cell signaling inhibitors at transcript levels as detected by qPCR. GAPDH was used as an endogenous control in qPCR. (e) Secreted chemokine levels CCL17 (M2a) and CCL1 (M2b) post Hz phagocytosis (24 h) from culture supernatant (24 h) in the presence of cell signaling inhibitors estimated by ELISA.(f) The relative MFI of CD206 (M2) of Hz‐fed MO in the presence of inhibitors as determined by flow cytometry and a representative overlay of treated cells with percentage positive MO are shown. (g) Depicting arginase activity and (h) nitric oxide release as detected by colorimetric assays in the presence of Hz and signaling inhibitors. (i) Intracellular reactive oxygen species production in Hz‐fed MO, treated with signaling inhibitors as detected by flow cytometry. Representative overlay depicting the same is shown. (j) Inhibition of lymphocyte proliferation measured by (^3^H) Thymidine incorporation in Hz‐fed MO, treated with signaling inhibitors. Results are *M* ± SEM from at least three independent donors. Significance levels: **p < 0.01, ***p < 0.001 in comparison with the control as determined by one‐way ANOVA (Bonferroni test)

The role of p38 MAPK, PI3K‐AKT, and NF‐κB pathways has been implicated in IL‐10 synthesis and M2 polarization; however, their relevance in Hz‐induced M2‐like phenotype is not reported. In this study, exposure to Hz led to a significant surge in the phosphorylation status of p38 MAPK (pP38‐Y182), PI3K‐AKT (pAKT‐S473), and NF‐κB (p P65‐S276) in MO, indicating the activation of these pathways. Moreover, the decrease in IκBα further confirmed the activation of NF‐κB (Figure 2a and Supporting Information Figure S7). Furthermore, pharmacological inhibitors of these pathways, SB203580 (p38 MAPK), parthenolide (NF‐κB p65), and LY294002 (PI3K‐AKT) dramatically down‐regulated the expression and secretion of IL‐10 (Figure 2b,c), implicating the involvement of these pathways in Hz‐induced IL‐10 production in MO. Furthermore, we observed significant attenuation of M2‐associated CCL17 and CCL1 at the transcript and secreted levels in Hz‐fed MO pretreated with SB203580 (SB), parthenolide (PAR), and LY294002(LY) (Figure 2d,e). Moreover, decrease in surface expression of CD206 with reduction in percent positive cells and MFI corroborates the results of chemokine profiles confirming attenuation of M2‐like phenotype by the inhibitors (Figure 2f). The levels of TNF‐α, IL‐1β, and IL‐6 were markedly reduced with Hz in the presence of SB203580 and parthenolide. In contrast, IL‐1β and IL‐6 cytokines were further elevated in presence of LY294002 (Supporting Information Figure S8a,b). LY294002 can act on other pathways along with PI3K kinases that may be responsible for the regulation of IL‐6 and IL‐1β (Gharbi et al., 2007). To confirm the role of PI3K‐AKT pathway, we used another inhibitor—wortmannin in similar experiments. Wortmannin effectively reduced the level of IL‐1β, IL‐6, and IL‐10 in supernatants of MO fed with Hz, signifying the role of PI3K‐AKT pathway in inhibiting M2‐like phenotype (Supporting Information Figure S8c).

Establishing the role of p38 MAPK, PI3K‐AKT, and NF‐κB pathways in Hz‐driven MO activation toward M2‐like phenotype encouraged us to evaluate M2‐specific biochemical and functional properties in the presence of specific inhibitors. The enhanced effect of Hz on arginase activity was abrogated with inhibitors to p38 MAPK‐AKT, PI3K, and NF‐κB pathways (Figure 2g). Moreover, blocking of these pathways prevented the Hz‐induced inhibition of NO (Figure 2h) and ROS (Figure 2i) production. Surprisingly, LY294002 did not affect the ROS levels reduced by Hz (Figure 2i). It is noteworthy that proliferation of PHA‐stimulated lymphocytes cultured with Hz‐fed MO was restored in the presence of SB203580, parthenolide, and LY294002 (Figure 2j). These results strongly implicate the role of p38 MAPK, PI3K‐AKT, and NF‐κB pathways in Hz‐induced skewing of MO toward M2‐like phenotype and suppression of mitogen‐stimulated proliferation of lymphocytes.

### 3.3 Antimalarial drugs—ART and CHQ partial reverse Hz‐induced M2‐like phenotype

**Figure 3:**
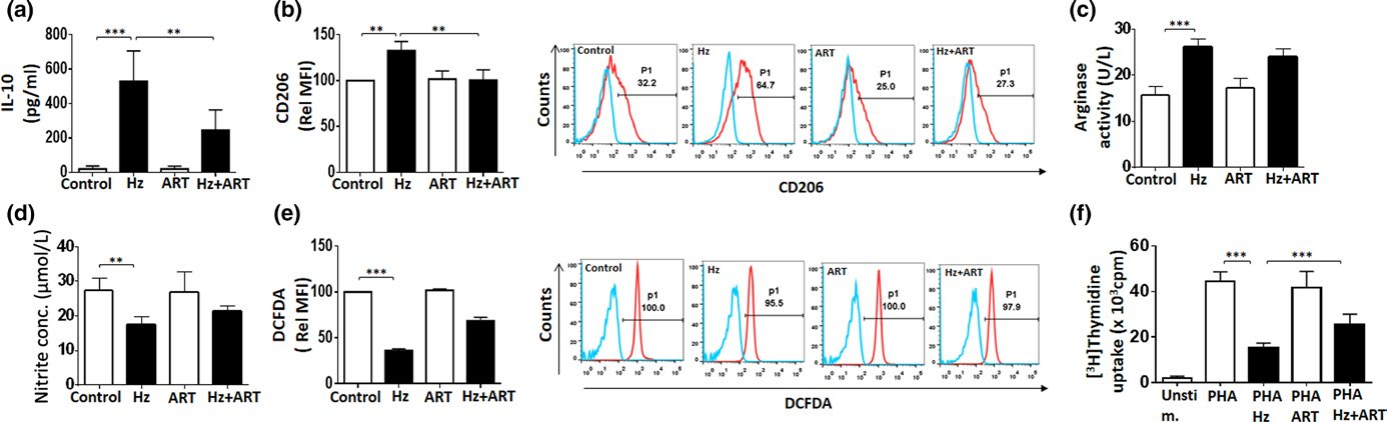
Artemisinin (ART) partially reverses hemozoin (Hz)‐induced M2‐associated phenotypic characters in monocytes (MO). Hz‐fed MO assessed for expression of M2‐related phenotypic markers—(a) secreted IL‐10 using ELISA, (b) CD206 by flow cytometry—in the presence of ART. A representative overlay of treated cells with percentage positive MO is shown. (c) Arginase activity (M2) and (d) nitric oxide (M1) release detected by colorimetric assays in Hz‐fed MO post ART treatment. (e) Intracellular reactive oxygen species (ROS) (M1) as monitored by flow cytometry post ART treatment in Hz‐fed MO. Relative MFI of DCFDA and representative overlays with percentage positive MO for ROS are shown. (f) Inhibition of lymphocyte proliferation measured by (^3^H) Thymidine incorporation in Hz‐fed MO, treated with ART. Results are *M* ± SEM from at least three independent donors. Significance levels:*p < 0.05, **p < 0.01, ***p < 0.001 in comparison with the control as determined by one‐way ANOVA (Bonferroni test)

**Figure 4:**
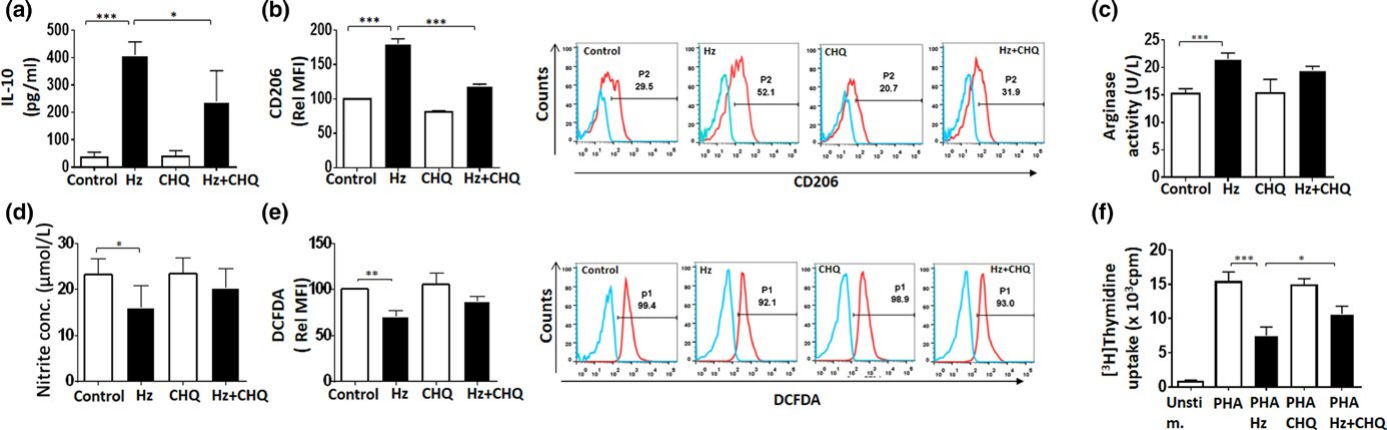
Chloroquine (CHQ) partially reverses hemozoin (Hz)‐induced M2‐associated phenotypic characters in monocytes (MO). Hz‐fed MO assessed for expression of M2‐related phenotypic markers—(a) secreted IL‐10 using ELISA, (b) CD206 by flow cytometry—in the presence of CHQ. A representative overlay of treated cells with percentage positive MO are shown. (c) Arginase activity (M2) and (d) nitric oxide (M1) release detected by colorimetric assays in Hz‐fed MO post CHQ treatment. (e) Intracellular reactive oxygen species (ROS) (M1) as monitored by flow cytometry post CHQ treatment in Hz‐fed MO. Relative MFI of DCFDA and representative overlays with percentage positive MO for ROS are shown. (f) Inhibition of lymphocyte proliferation measured by (^3^H) Thymidine incorporation in Hz‐fed MO, treated with CHQ. Results are *M* ± SEM from at least three independent donors. Significance levels:*p < 0.05, **p < 0.01, ***p < 0.001 in comparison with the control as determined by one‐way ANOVA (Bonferroni test)

Antiparasitic activity of ART and CHQ coupled with the reported immunomodulatory roles suggests their possible effect on the activation of MO toward M2‐like phenotype during malaria pathogenesis. The concentrations of drugs used in the current experiments were based on previous studies on MO (Jang, Choi, Byun, & Jue, 2006; Wang et al., 2011). ART in concentrations ranging from 5 to 400 μM did not affect the viability of MO as assessed for cytotoxicity by MTT assay (Supporting Information Figure S9a). In our study, we found that treatment of MO preexposed to Hz with ART resulted in dramatic reduction of IL‐10 levels (Figure 3a) and expression of the surface marker CD206 in terms of percent positive cells and MFI (Figure 3b). ART also exhibited the ability to significantly diminish secretion of other M2 cytokines—CCL17, CCL1, IL‐6, TNF‐α, and IL‐1β (Figure S9b)—but did not significantly alter arginase activity, NO, and ROS production (Figure 3c–e). Importantly, ART partially reversed Hz‐induced immunosuppression as demonstrated by partial restoration of mitogen‐stimulated lymphocyte proliferation (Figure 3f). ART in the dose range of 5–20 μM decreased secretion of phenotypic markers IL‐10, CCL17, CCL1, and CD206 surface expression in Hz‐fed MO (Supporting Information Figure S9c). The Hz‐induced NF‐κB activation was inhibited by ART as indicated by decreased p65 phosphorylation and increased IκBα in MO (Supporting Information Figure S9d). These results suggest the contribution of NF‐κB in inhibition of M2 phenotypic markers by ART. CHQ showed similar effect on the Hz‐driven M2‐like phenotype in MO, inhibited NF‐κB p65 phosphorylation, and increased IκBα levels (Figure 4a–f and Supporting Information Figure S10). Taken together, these results suggest that ART and CHQ treatment partially reverse Hz‐induced M2‐like MO activation.

## 4 DISCUSSION

*P. falciparum* infection manifests itself as varying degrees of severity in malaria, inclusive of uncomplicated, mild, severe, and cerebral malaria. Variable susceptibility to the parasite is governed by a constant conflict between immunoprotection and immunopathology. The immune system acts like a double‐edged sword, wherein interplay among anti‐ and pro‐inflammatory responses dictates disease outcome. Hence, studies on interactions between host and parasite components are important to provide a better understanding of factors affecting directionality of immune response. MO, an important constituent of mononuclear phagocytic system, plays an important role during parasite infection. Parasite components, including Hz, released during the erythrocytic cycle are readily taken up by patrolling MO. The ingested Hz is accumulated and affects the accessory functions of MO and other immune cells, which in‐turn may contribute to immunosuppression seen in malaria. We show here that Hz drives MO toward M2‐like phenotype and is mediated by PI3K‐AKT, NF‐κB, and p38 MAPK signaling pathways. Antimalarial drugs—CHQ and ART—partially reversed Hz‐induced M2‐like phenotype, thereby supporting their immunomodulatory role.

Blood mononuclear phagocytes display plasticity and have the ability to polarize toward M1 or M2 phenotypes in response to environmental stimuli. Findings from our study demonstrate that Hz drives human MO toward M2‐like phenotype, as characterized by the expression of phenotypic markers including elevated IL‐10, mannose receptor expression CD206, and arginase activity. Hz‐induced M2‐like MO comprise of M2a and M2b subtypes based on the expression of CCL17 and CCL1. Since chemokines, CCL17 and CCL1, function as agonists for CCR4 and CCR8 receptors, respectively (Mantovani et al., 2004), it is likely that Hz might facilitate recruitment of CCR4 and CCR8‐bearing TH2 cells and regulatory T cells of TH2 immunity which contribute to immunosuppression. It is noteworthy in this context that regulatory T cells suppress T‐cell responses in malaria (Hisaeda et al., 2004). Furthermore, Hz induces production of M2b‐specific cytokines—TNF‐α, IL‐1β, and IL‐6 in human MO. These data corroborate with reports demonstrating the presence of inflammatory cytokines IL‐1β, TNF‐α, and IL‐6 along with high level of anti‐inflammatory IL‐10 in plasma of malaria patients (Perera et al., 2013; Weinberg et al., 2016).

Functionally, M1‐type MO are characterized by elevated production of NO and ROI that are essential for clearance of pathogens (Mantovani et al., 2004). Conversely, M2‐type MO express high arginase activity which is due to production of arginase‐1, the enzyme that converts arginine to ornithine and urea causing depletion of arginine and reduction of NO (Biswas & Mantovani, 2012; Galván‐Peña & O'Neill, 2014; Martinez & Gordon, 2014). In malaria, depletion of NO results in low NO bioavailability that affects adherence of parasites to endothelium and is associated with severity of the disease. High arginase activity and an inverse relation between disease severity and NO production are recently reported in malaria (Weinberg et al., 2016). In this context, MO fed with Hz demonstrate elevated arginase activity compared with untreated and latex‐ingested MO. These data along with the findings from studies that *plasmodium* parasites (Olszewski et al., 2009) and lysed infected erythrocytes (Reiter et al., 2002) release arginase suggest that cumulative elevation of arginase activity from these sources might contribute to M2‐like activity. Recent studies have reported Hz‐induced production of ROS (Jaramillo et al., 2005) and NO (Jaramillo, Gowda, Radzioch, & Olivier, 2003) in MO. In contrast to these reports, we found that Hz significantly reduced production of NO and ROS levels compared with untreated and latex‐ingested MO. The difference in the results may be attributed to the nature of Hz used; while the earlier studies were performed with delipidized Hz and β‐hematin, our experiments were conducted with natural Hz. In addition, mitogen‐stimulated lymphocyte proliferation was significantly suppressed in the presence of Hz‐fed MO compared with control and latex‐ingested MO. This observation is in agreement with the study that malaria patients have suppressed T‐ and B‐cell functions (Zander & Butler, 2013).

The signaling pathways p38 MAPK, PI3K‐AKT, and NF‐κB have been implicated in IL‐10 synthesis (Saraiva & O'Garra, 2010) and M2 polarization (Zhang et al., 2010)^‐^(Liu et al., 2017). In this study, Hz activated p38 MAPK, PI3K‐AKT, and NF‐κB pathways and pharmacological inhibitors for these pathways dramatically down‐regulated the expression and secretion of IL‐10, thus, over‐riding the induction triggered by Hz exposure. In line with this observation, the inhibitors effectively reduced Hz‐induced M2‐like phenotypic, biochemical, and functional properties. While these findings implicate the role of p38 MAPK, PI3K‐AKT, and NF‐κB pathways, we cannot rule out the possibility of other signaling pathways in Hz‐driven activation of MO toward M2 phenotype. Since Hz does not induce a specific phenotype subset but drives the MO toward M2a and M2b types, it may be postulated that Hz stimulates atypical activation mediated through PI3K‐AKT, p38 MAPK, and NF‐κB pathways.

We next aimed to identify strategies that could aid in reversing the effect of Hz in driving the MO to an M2‐like phenotype. In this regard, we conducted experiments with CHQ and ART for three reasons; first, they are reported to inhibit pathways crucial for polarization to M2 type; second, they are widely used for treatment of malaria; and third, they possess immunomodulatory properties. ART have been reported to decrease IL‐10, IL‐1β, IL‐6, and TNF‐α levels (Shakir, Hussain, Javeed, Ashraf, & Riaz, 2011; Wu et al., 2016) through MAPK (Wang et al., 2009), NF‐κB (Prato, Gallo, Giribaldi, Aldieri, & Arese, 2010; Wang et al., 2011), and PI3K‐AKT (Kim et al., 2013) pathways. CHQ has also been demonstrated to act through MAPK (Kono et al., 2008; Weber, Chen, & Levitz, 2002) and NF‐κB(Long, Liu, Wang, Zhou, & Zheng, 2015; Yang et al., 2013) pathways and decrease IL‐1β, IL‐6, and TNF‐α in mononuclear phagocytes in different diseases (Jang et al., 2006; Long et al., 2015). In another study, CHQ has been shown to be an effective anticancer drug in mice by inhibiting tumor resistant MQ (M2 MQ) and decreasing TGF‐β and IL‐10 production. The effect was accompanied by decreased myeloid‐derived suppressor cells (MDSC), Tregs, and increasing CD8^+^ T cells in tumor milieu (Zhang et al., 2017). The ability of these drugs to inhibit crucial pathways and production of cytokines/chemokines that are signature for M2 type MO has extended their use for other parasitic diseases, rheumatic diseases, and cancer. In our study, the drugs were found to be more effective in reducing the expression of phenotypic markers—cytokines, chemokines, and surface marker—CD206 associated with M2‐like phenotype than reversing altered biochemical and functional properties. ART did not reverse arginase activity in Hz‐fed MO. Our finding is supported by an earlier report that ART did not significantly alter arginase activity in *leishmania donovani*‐infected MQ though it enhanced protective immune responses by reversing NO levels (Sen, Ganguly, Saha, & Chatterjee, 2010). These observations are not surprising as CHQ and ART are known for their antimalarial action through multiple modes on their target, but their mechanism of action as immunomodulators is not clearly elucidated. The M2 phenotype is previously related to abnormal autophagy and angiogenesis (Li et al., 2016). We speculate that CHQ, a known autophagy inhibitor, may attenuate Hz‐induced M2‐like MO activation by inhibiting autophagy. Previously, *falciparum* malaria pathogenesis has been related to upregulated angiogenic factors VEGF, Ang‐2, and sFLT‐1 along with dysregulated and excessive immune responses (Muehlenbachs, Mutabingwa, Edmonds, Fried, & Duffy, 2006; Silver, Higgins, McDonald, & Kain, 2010). The antiparasitic action of ART is accompanied with disruption of parasite proteins, alteration of mitochondrial functions, and angiogenesis (Ak, 2015; Shakir et al., 2011). In relation to ability of M2 phenotype to stimulate angiogenesis, ART may attenuate Hz‐induced M2‐like phenotype by altering the release of angiogenic factors.

**Figure 5:**
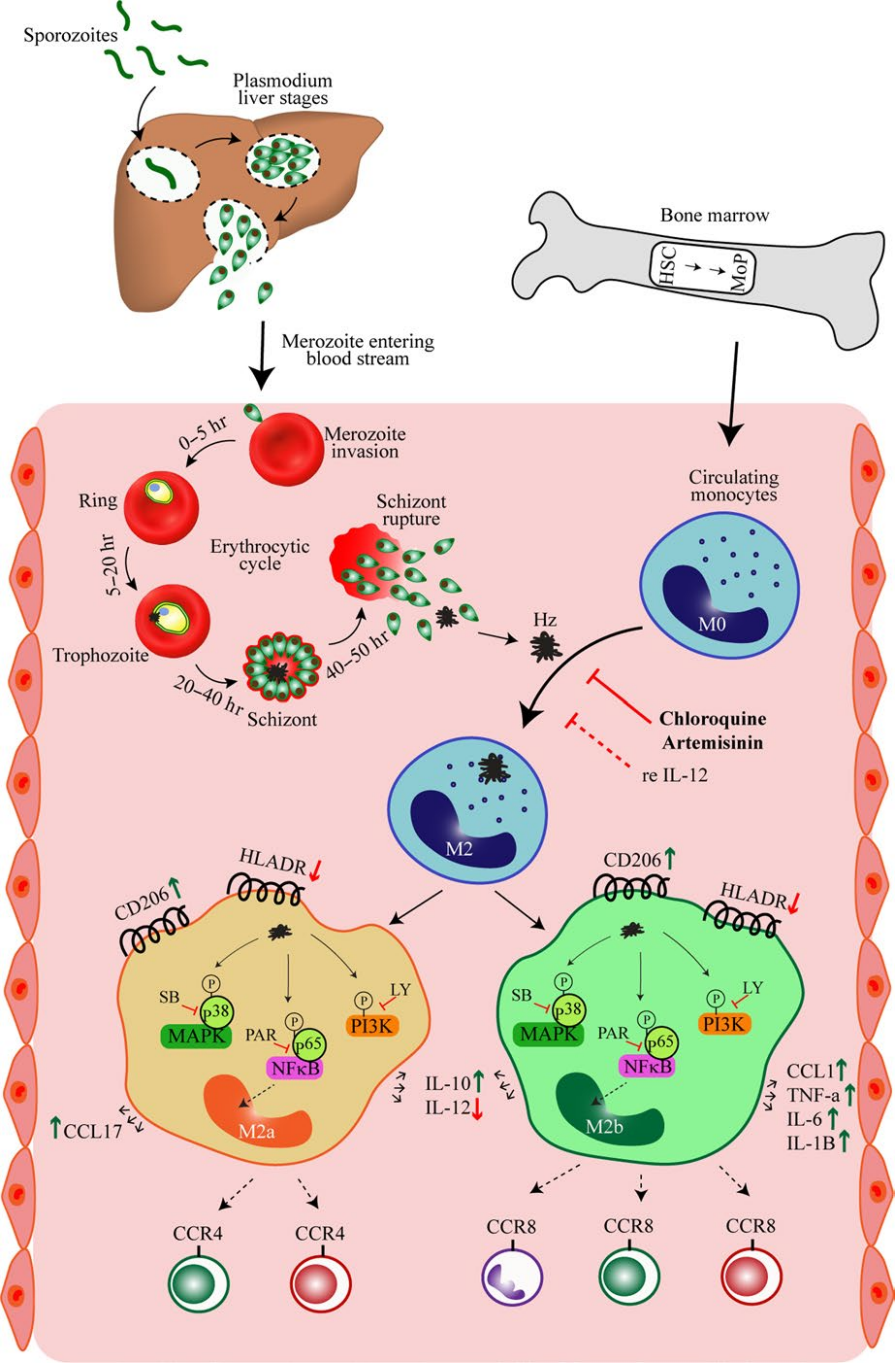
Proposed model for hemozoin (Hz)‐induced M2‐like monocytes (MO) during infection. Hz released after schizont rupture is ingested by circulating MO in blood, activates PI3K‐AKT, NF‐κB, and p38 MAPK pathways, resulting in increased expression of M2 markers IL‐10, CD206, arginase activity. Specifically, Hz induces M2a (CCL1) and M2b (CCL17, TNF‐α, IL‐1β, and IL‐6) phenotype that regulate TH2 responses. Hz attenuates HLA‐DR expression, nitric oxide production, and reactive oxygen species—features of M1 phenotype. Antimalarial drugs—artemisinin and chloroquine—partially reverse the Hz‐induced M2‐like phenotype

The schematic diagram (Figure 5) summarizes the Hz‐induced alterations in M1‐ and M2‐associated markers, the pathways involved and partial reversal by CHQ and ART in human MO. Hz released after schizont rupture is readily ingested by circulating MO in blood; activates PI3K‐AKT, NF‐κB, and p38 MAPK pathways resulting in increased expression of M2 markers; and drives the MO toward M2 (M2a and M2b)‐like phenotype. Antimalarial drugs—ART and CHQ—partially reverse the process of activation of MO toward M2‐like phenotype. In conclusion, the findings from this study add a new dimension to the understanding of mechanism/s underlying Hz‐induced immunosuppression by demonstrating its ability to drive the MO toward M2‐like phenotype. Though antimalarial resistance poses a major challenge and discourages the continuation of CHQ and ART, we propose that these drugs may be re‐examined for their potential as immunomodulators and candidates for adjunctive treatment in malaria.

## AUTHOR CONTRIBUTION

D.B. performed research experiments, analyzed data, and wrote the paper; A.K. and M.D. performed experiments; P.D. conceived, designed, supervised, performed research experiments, analyzed, and wrote the paper; P.S., conceived, designed, analyzed, and wrote the paper.

## CONFLICT OF INTEREST

Research was conducted in the absence of any commercial or financial relationships that could be construed as a potential conflict of interest.
