# Supplementary figures and images for: Hemozoin‐induced activation of human monocytes toward M2‐like phenotype is partially reversed by antimalarial drugs—chloroquine and artemisinin
Source: Microbiologyopen. 2018 Jun 7;8(3):e00651. doi: 10.1002/mbo3.651 (PMC6436431; doi:10.1002/mbo3.651)

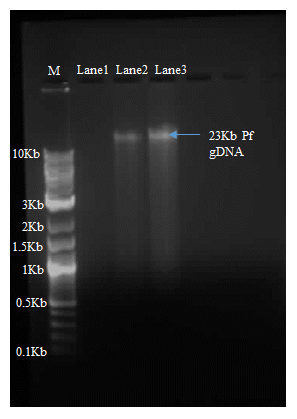

Supplement: Supplementary file 1 [file MBO3-8-e00651-s001.tif]

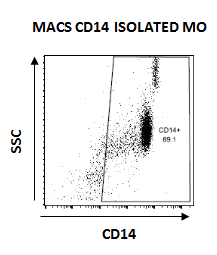

Supplement: Supplementary file 2 [file MBO3-8-e00651-s002.tif]

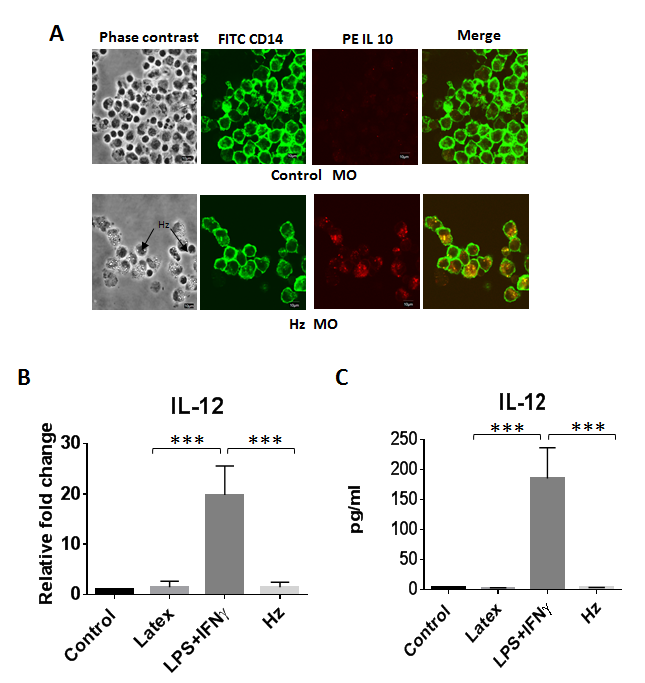

Supplement: Supplementary file 3 [file MBO3-8-e00651-s003.tif]

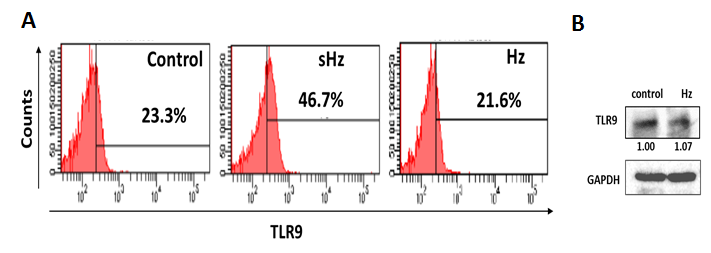

Supplement: Supplementary file 4 [file MBO3-8-e00651-s004.tif]

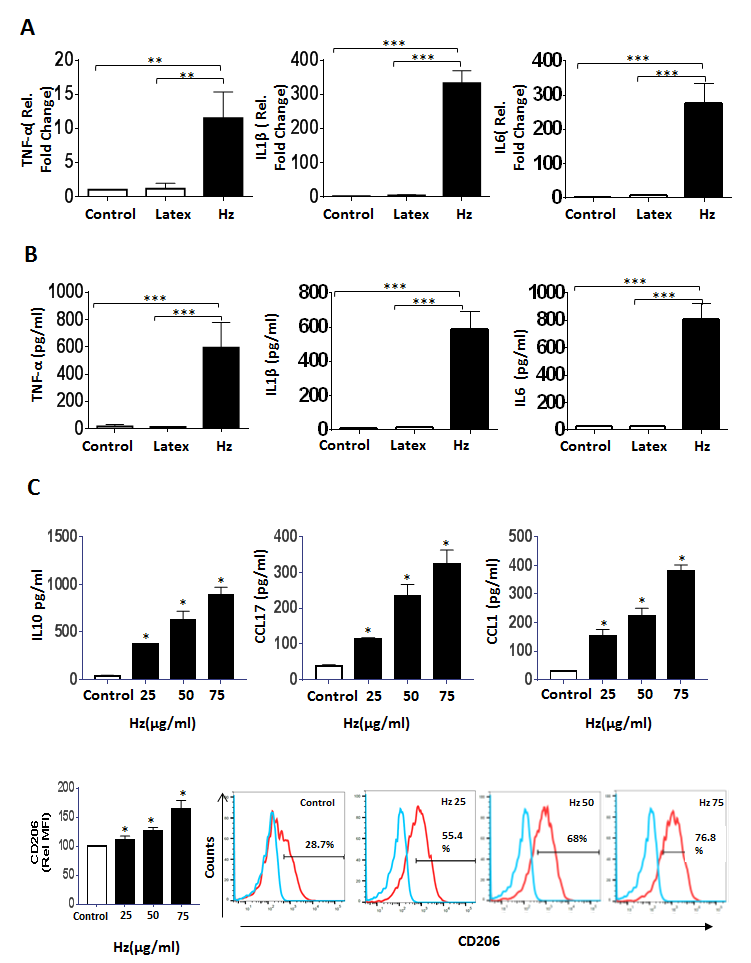

Supplement: Supplementary file 5 [file MBO3-8-e00651-s005.tif]

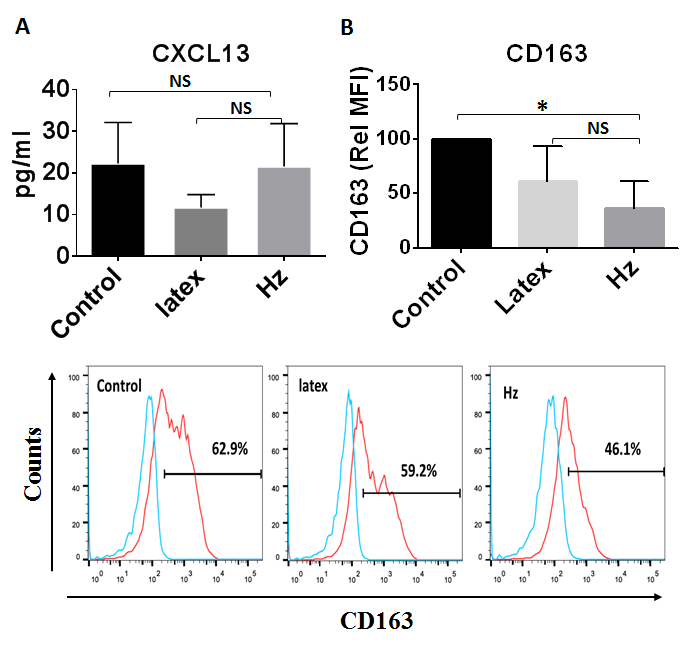

Supplement: Supplementary file 6 [file MBO3-8-e00651-s006.tif]

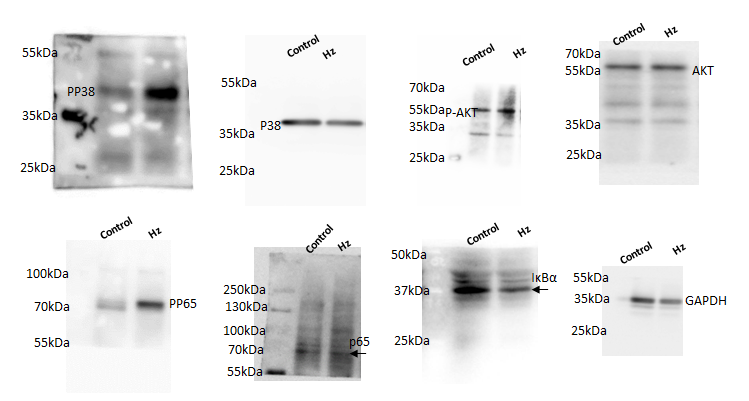

Supplement: Supplementary file 7 [file MBO3-8-e00651-s007.tif]

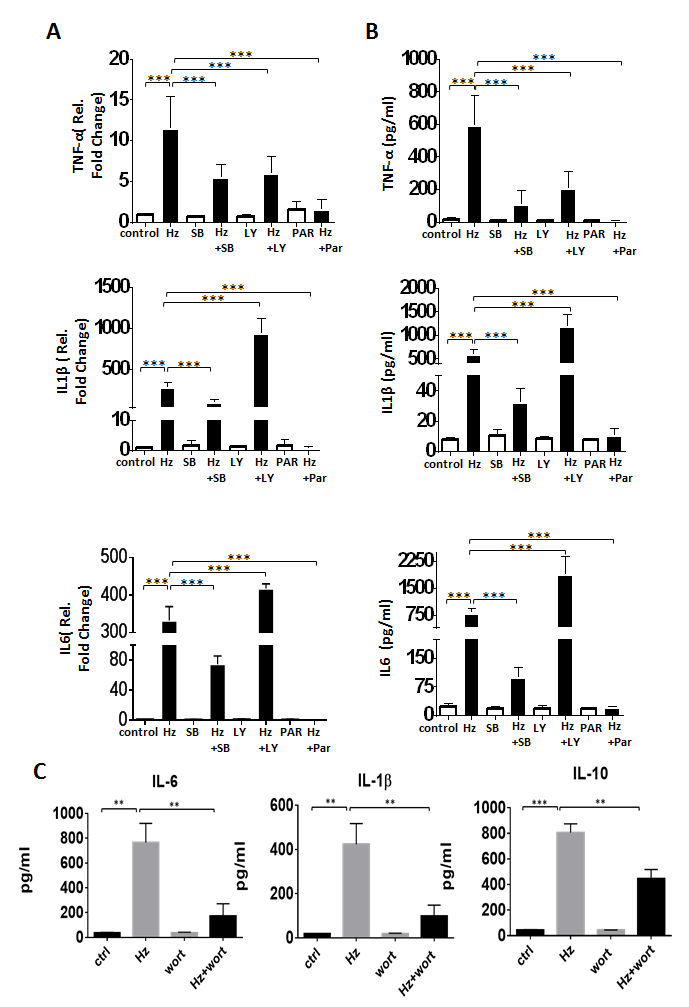

Supplement: Supplementary file 8 [file MBO3-8-e00651-s008.tif]

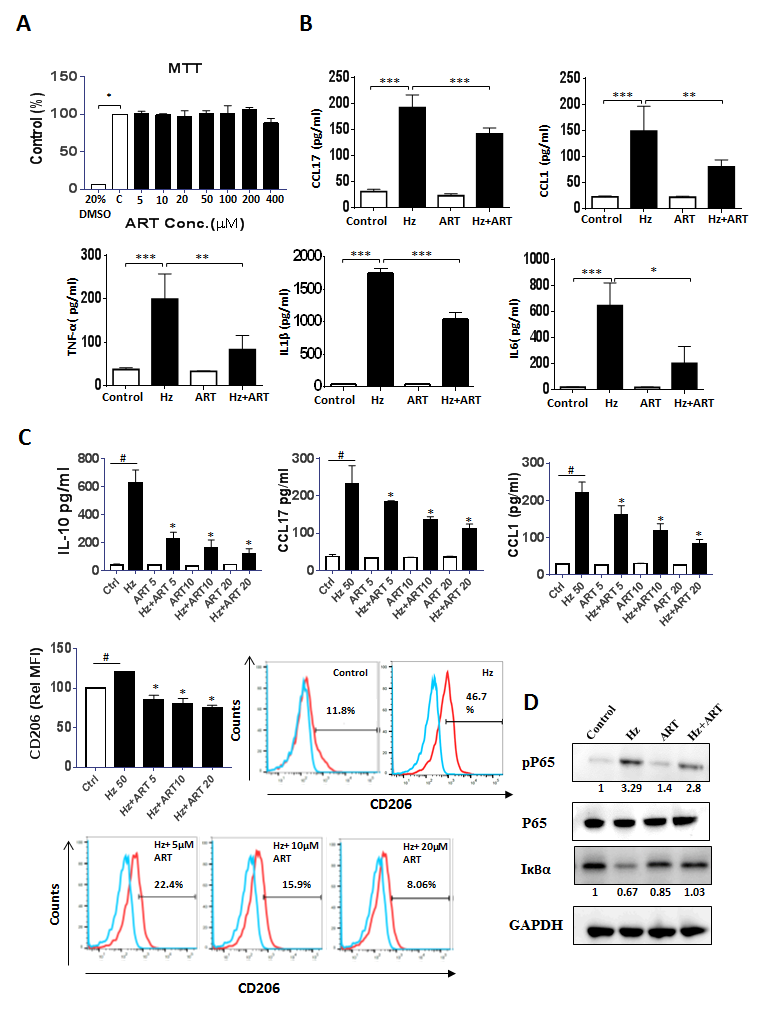

Supplement: Supplementary file 9 [file MBO3-8-e00651-s009.tif]

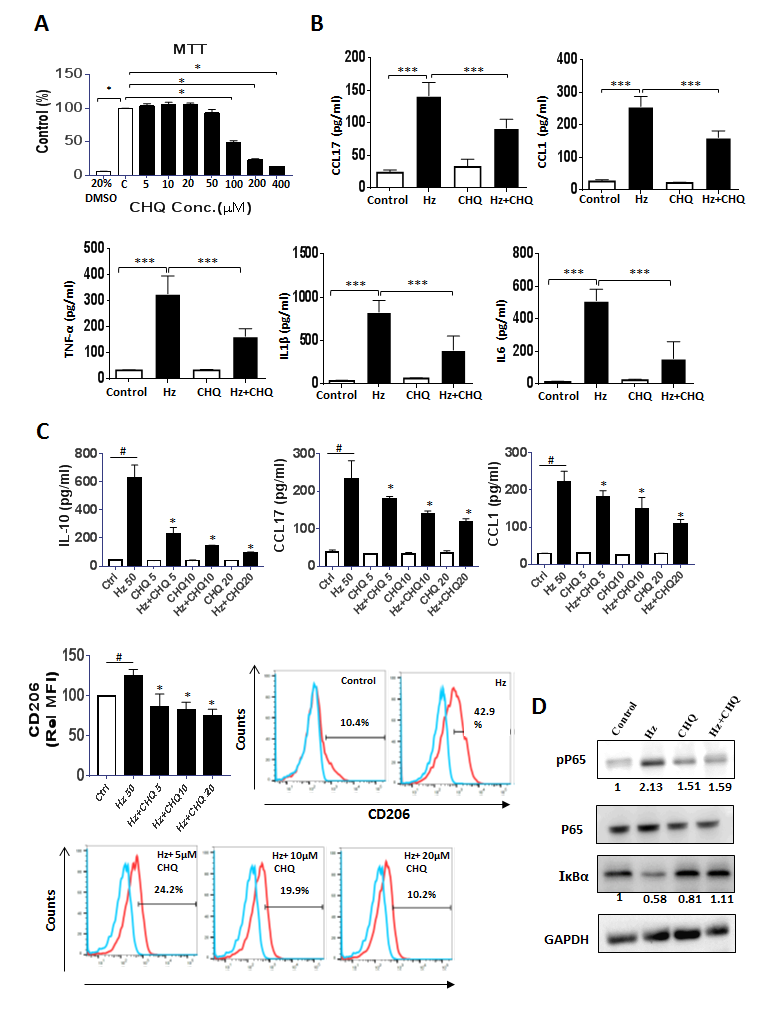

Supplement: Supplementary file 10 [file MBO3-8-e00651-s010.tif]
