# Supplementary material for: Hemozoin‐induced activation of human monocytes toward M2‐like phenotype is partially reversed by antimalarial drugs—chloroquine and artemisinin
Source: Microbiologyopen. 2018 Jun 7;8(3):e00651. doi: 10.1002/mbo3.651 (PMC6436431; doi:10.1002/mbo3.651)
Supplement: Supplementary file 11 [file MBO3-8-e00651-s011.docx]

**Supplementary Table S1 List of primers used in Quantitative RT-PCR**

| **Primer name** |  | **sequence** |
| --- | --- | --- |
| CCL1 | Forward | 5' -CTTGCTGCTAGCTGGGATGT -3' |
|  | Reverse | 5' -CTTGAATATTAAGCCCTCATTGGAG-3' |
| CCL17 | Forward | 5' -AGGGATGCCATCGTTTTTGTAA -3' |
|  | Reverse | 5' -GCTTCAAGACCTCTCAAGGCT -3' |
| IL10 | Forward | 5' -GGCACCCAGTCTGAGAACAG -3' |
|  | Reverse | 5' -TGGCAACCCAGGTAACCCTTA-3' |
| IL12p70 | Forward | 5' -GCTCCAGAAGGCCAGACAAA -3' |
|  | Reverse | 5' -GCCAGGCAACTCCCATTAGT-3' |
| TNF alpha | Forward | 5' -CCCATGTTGTAGCAAACCCTC-3' |
|  | Reverse | 5' -TATCTCTCAGCTCCACGCCA-3' |
| IL-6 | Forward | 5' -CAATGAGGAGACTTGCCTGG-3' |
|  | Reverse | 5' -TGGGTCAGGGGTGGTTATTG-3' |
| IL-1beta | Forward | 5' -CATTGCTCAAGTGTCTGAAGC6 -3' |
|  | Reverse | 5' -GTGGTGGTCGGAGATTCGTA-3' |
| GAPDH | Forward | 5' -AGCCGCATCTTCTTTTGCGT -3' |
|  | Reverse | 5' -GACCAAATCCGTTGACTCCGAC -3' |
