# Supplementary material for: Hemozoin‐induced activation of human monocytes toward M2‐like phenotype is partially reversed by antimalarial drugs—chloroquine and artemisinin
Source: Microbiologyopen. 2018 Jun 7;8(3):e00651. doi: 10.1002/mbo3.651 (PMC6436431; doi:10.1002/mbo3.651)
Supplement: Supplementary file 12 [file MBO3-8-e00651-s012.docx]

**Supporting Information**

**Hemozoin-induced activation of human monocytes towards M2-like phenotype is partially reversed by anti-malarial drugs-chloroquine and artemisinin**

**Deepali Bobade, Ashwin V Khandare, Mangesh Deval, Padma Shastry**, D. Prakash***

National Centre for Cell Science (NCCS), Ganeshkhind, Pune, India

**Table of Contents:**

**1 supplementary Table**

**10 Supplementary Figures**

**Supplementary Table S1 List of primers used in Quantitative RT-PCR**

| **Primer name** |  | **sequence** |
| --- | --- | --- |
| CCL1 | Forward | 5' -CTTGCTGCTAGCTGGGATGT -3' |
|  | Reverse | 5' -CTTGAATATTAAGCCCTCATTGGAG-3' |
| CCL17 | Forward | 5' -AGGGATGCCATCGTTTTTGTAA -3' |
|  | Reverse | 5' -GCTTCAAGACCTCTCAAGGCT -3' |
| IL10 | Forward | 5' -GGCACCCAGTCTGAGAACAG -3' |
|  | Reverse | 5' -TGGCAACCCAGGTAACCCTTA-3' |
| IL12p70 | Forward | 5' -GCTCCAGAAGGCCAGACAAA -3' |
|  | Reverse | 5' -GCCAGGCAACTCCCATTAGT-3' |
| TNF alpha | Forward | 5' -CCCATGTTGTAGCAAACCCTC-3' |
|  | Reverse | 5' -TATCTCTCAGCTCCACGCCA-3' |
| IL-6 | Forward | 5' -CAATGAGGAGACTTGCCTGG-3' |
|  | Reverse | 5' -TGGGTCAGGGGTGGTTATTG-3' |
| IL-1beta | Forward | 5' -CATTGCTCAAGTGTCTGAAGC6 -3' |
|  | Reverse | 5' -GTGGTGGTCGGAGATTCGTA-3' |
| GAPDH | Forward | 5' -AGCCGCATCTTCTTTTGCGT -3' |
|  | Reverse | 5' -GACCAAATCCGTTGACTCCGAC -3' |

**
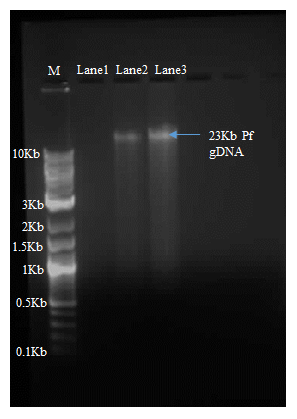
**

**S1 Fig. Purity of natural Hz.** Agarose gel electrophoresis (0.8% agarose) of DNA isolated from *Plasmodium falciparum* natural Hz or parasite culture by phenol-chloroform method. Lane M, 100 kb DNA size marker, Lane 1, 50µg nHz. Lane 2-500ng parasite DNA, Lane 3-1µg parasite DNA used as standard.


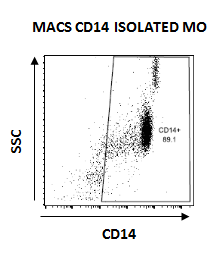


**S2 Fig. Purity of CD14^+^ human MO used for the experiments.**

The dot plot depicts representative figure of MO purity, isolated by CD14^+^ Magnetic associated cell sorting (MACS) from human PBMC.

**
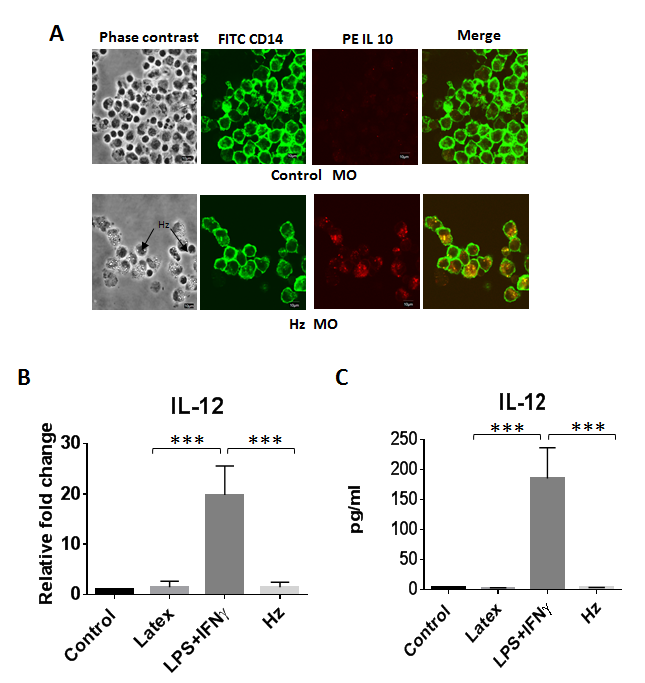
**

**S3 Fig. Expression of IL-10 (intracellular) and IL-12 (transcript and secreted) levels in Hz treated MO.**

**(A)** M2 MO were confirmed by dual staining for membranous CD14 and intracellular IL-10. Scale, 10µm. **(B)** MO exposed to LPS (1µg/ml) and IFNγ (20ng/ml), Hz and latex were assessed for cytokine IL-12p70 (M1) at transcript level (12h) as detected by qPCR **(C)** and secreted (24h) in culture supernatants by ELISA.

**
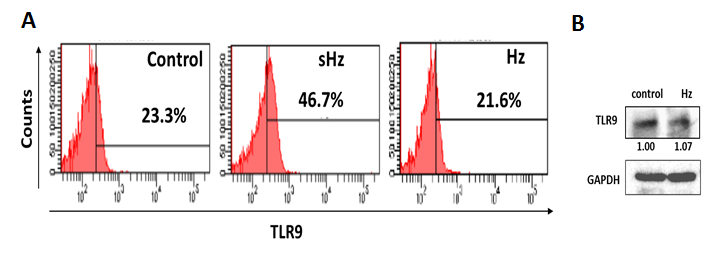
**

**S4 Fig. Expression of TLR9 in Hz treated MO.**

The surface molecule expression of TLR9 as determined by flow-cytometry (A) and protein levels by western blotting (B) in human MO exposed to sHz and natural Hz for 2h followed by incubation of 24h. Fold changes obtained by densitometry on normalizing with GAPDH are indicated below the blot.


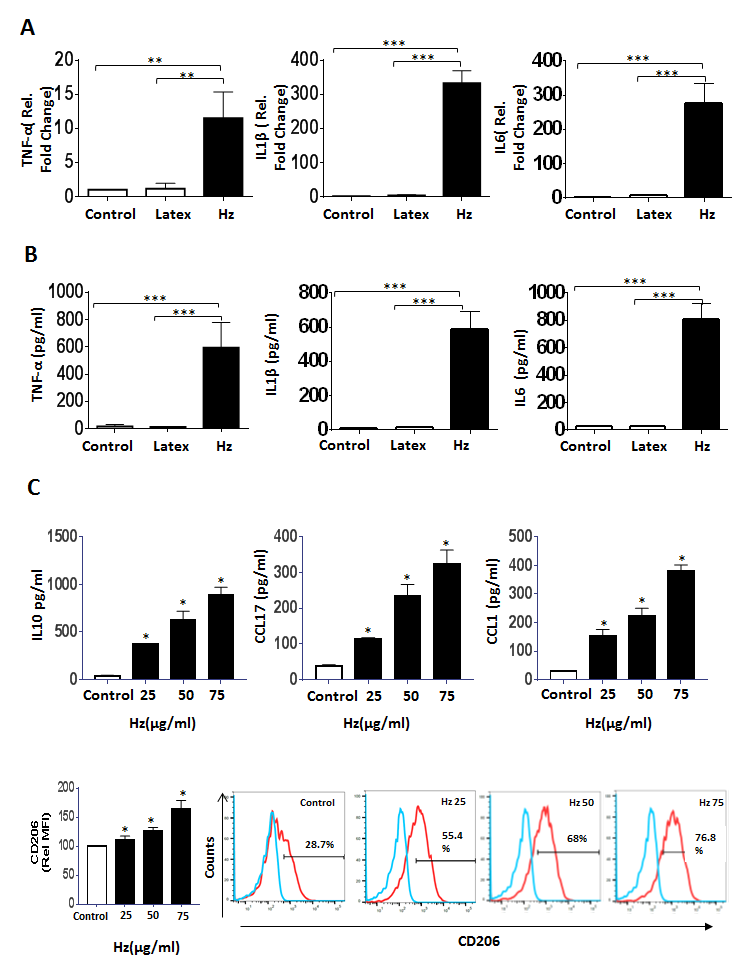


**S5 Fig. Expression and secretion of accessory M2 markers in Hz fed MO.**

Hz fed MO were assessed for cytokines TNF-α, IL-1β and IL-6 (M2b) at transcript level (12h) as detected by qPCR **(A)** and secreted cytokines (24h) in culture supernatants by ELISA **(B)**. GAPDH was used as an endogenous control in qPCR. Latex beads were used a phagocytosis control. The data is represented as mean ± SEM from 3 individuals. **(C)** IL-10, CCL17 and CCL1 as assessed in culture supernatants of Hz (25-75µg/ml) fed MO by ELISA and surface expression of CD206 on MO by flow cytometry. Significance levels: **P<0.01, ***P < 0.001 in comparison with the control and latex as determined by One way ANOVA (Bonferroni test).


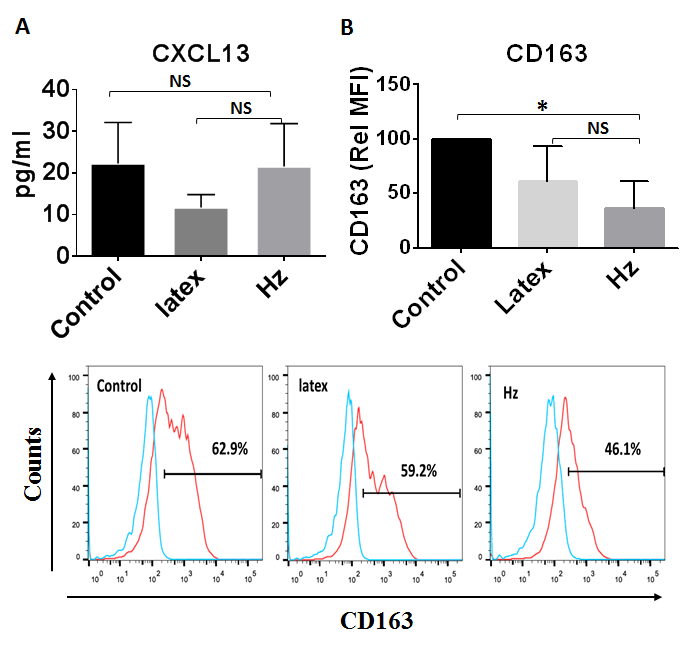


**S6 Fig. Effect of Hz on M2C markers** (A) Expression of CXCL13 at secreted protein levels in supernatants and (B) surface expression of CD163 on human MO exposed to latex, LPS+IFNγ and Hz for 24h as determined by ELISA and flow-cytometry respectively.*P>0.05.


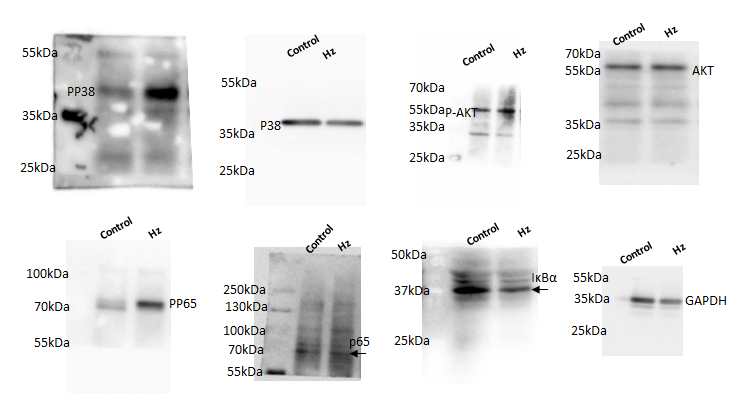


**S7 Fig. Full length blots from figure 2 (A).**

The original blot of Figure 2A. MO were fed with Hz (2h). Whole-cell lysates of untreated and Hz fed MO were analyzed by western blot using the appropriate antibodies. The specific protein bands were analyzed using the GE image quant 4000 chemiluminesence system (GE Healthcare Life Sciences). (Re-blot: The blots were stripped in EDTA solution and probed with either anti-p65-NF-κB, anti-AKT, anti-p38-MAPK, IκBα.)


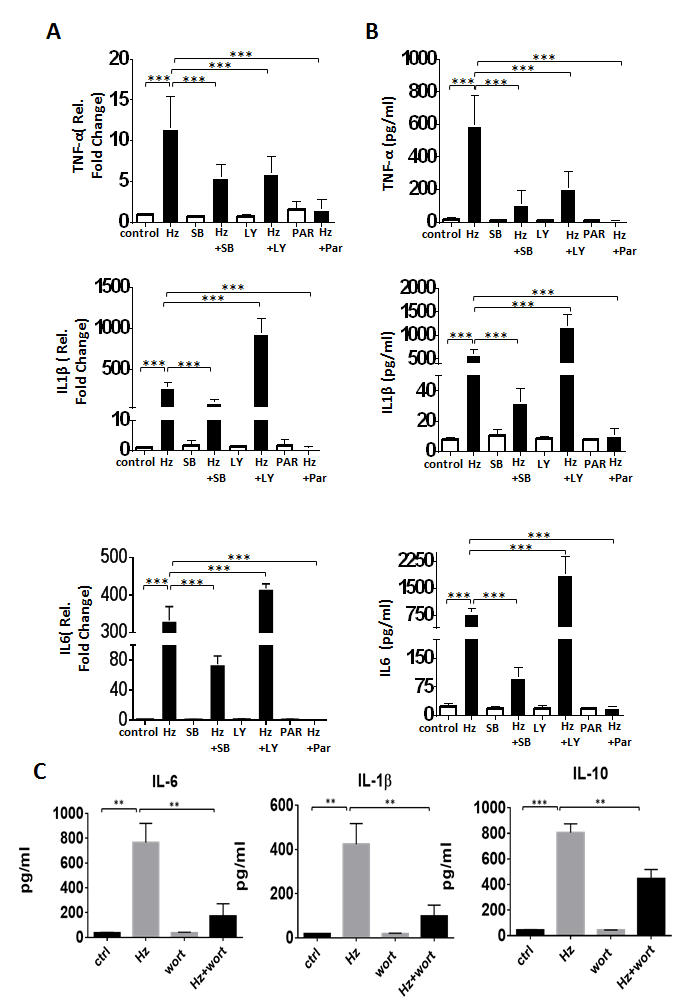


**S8 Fig. Effect of signaling inhibitors SB203580, Parthenolide and LY294002 on expression and secretion of accessory M2b markers in Hz fed MO.**

Expression of Hz induced TNF-α, IL-1β and IL-6 (M2b) in presence of cell signaling inhibitors SB203580 (SB), parthenolide (PAR), and LY294002 (LY) at transcript levels (12h) as detected by qPCR **(A)** and secreted chemokine levels (24h) as detected by ELISA **(B)**. Expression of cytokines IL-1β, IL-6 and IL-10 at secreted levels in supernatants of human MO pretreated with PI3K inhibitor wortmanin (50ng/ml) followed by exposure to Hz for 24h as determined by ELISA (C).The data represented is mean ± SEM from 3 individuals. Significance levels: **P < 0.01, ***P < 0.001 in comparison with the control and latex as determined by One way ANOVA (Bonferroni test).

**
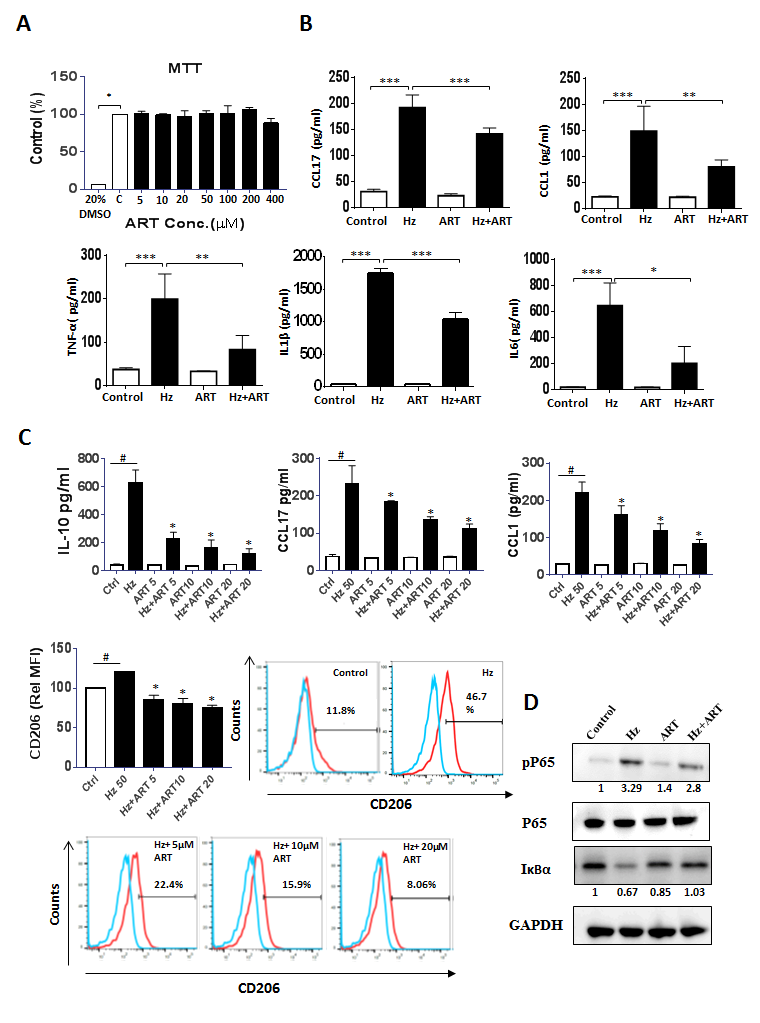
**

**S9 Fig. Effect of artemisinin on cyto-toxicity of MO, expression and secretion of M2a and M2b markers in Hz fed MO.**

1. Effect of ART (5-400µM) on viability of MO as assessed by MTT. (B) Hz fed MO assessed for expression of M2 related phenotypic markers – secreted CCL17 (M2a), CCL1, TNF-α, IL-1β and IL-6 (M2b) (24h) using ELISA– in presence of ART. (C) The secretion of cytokine IL-10, CCL17, CCL1 and surface expression of CD206 as detected by ELISA and flow cytometry in ART (5-20µM) post treated Hz fed MO. The data represented is mean ± SEM from more than 3 individuals. Significance levels: *P<0.05, **P < 0.01, ***P < 0.001 in comparison with the control and latex as determined by One way ANOVA (Bonferroni test). (D) Western blot analysis of whole-cell lysates from control and Hz fed MO in the presence or absence of ART (2h) for total and phosphorylated levels of NF-κB p65 and IκBα. Fold change obtained from densitometric analysis of phospho-p65-NF-κB, normalized with GAPDH are indicated above blots.


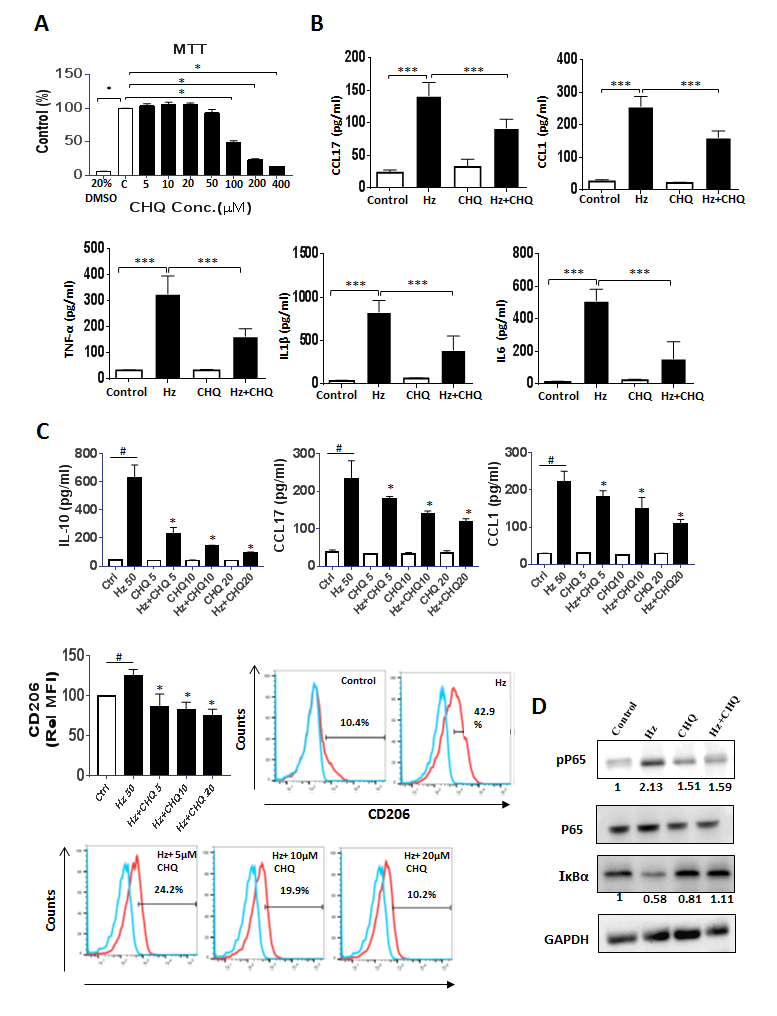


**S10 Fig. Effect of chloroquine on cyto-toxicity of MO, expression and secretion of M2a and M2b markers in Hz fed MO.**

(A) Effect of CHQ (5-400µM) on viability of MO as assessed by MTT. (B) Hz fed MO assessed for expression of M2 related phenotypic markers – secreted CCL17 (M2a), CCL1, TNF-α, IL-1β and IL-6 (M2b) (24h) using ELISA– in presence of CHQ. (C) The secretion of cytokine IL-10, CCL17, CCL1 and surface expression of CD206 as detected by ELISA and flow cytometry in CHQ (5-20µM) post treated Hz fed MO. The data represented is mean ± SEM from more than 3 individuals. Significance levels: *P<0.05, **P < 0.01, ***P < 0.001 in comparison with the control and latex as determined by One way ANOVA (Bonferroni test). (D) Western blot analysis of whole-cell lysates from control and Hz fed MO in the presence or absence of CHQ (2h) for total and phosphorylated levels of NF-κB p65 and IκBα. Fold change obtained from densitometric analysis of phospho-p65-NF-κB, normalized with GAPDH are indicated above blots.
